# The ABA intervention for improving breastfeeding initiation and continuation: Feasibility study results

**DOI:** 10.1111/mcn.12907

**Published:** 2019-12-02

**Authors:** Joanne L. Clarke, Jenny Ingram, Debbie Johnson, Gill Thomson, Heather Trickey, Stephan U. Dombrowski, Alice Sitch, Fiona Dykes, Max Feltham, Christine MacArthur, Tracy Roberts, Pat Hoddinott, Kate Jolly

**Affiliations:** ^1^ Institute of Applied Health Research University of Birmingham UK; ^2^ Centre for Academic Child Health University of Bristol UK; ^3^ Maternal and Infant Nutrition and Nurture Unit (MAINN) University of Central Lancashire UK; ^4^ DECIPHER, Department of Social Medicine Cardiff University UK; ^5^ Faculty of Kinesiology University of New Brunswick Canada; ^6^ Division of Psychology University of Stirling UK; ^7^ NIHR Birmingham Biomedical Research Centre University Hospitals Birmingham NHS Foundation Trust and University of Birmingham UK; ^8^ Birmingham Clinical Trials Unit University of Birmingham UK; ^9^ Health Economic Unit University of Birmingham UK; ^10^ Nursing, Midwifery and Allied Health Professions Research Unit University of Stirling UK

**Keywords:** assets‐based approach, behaviour change theory, breastfeeding, infant feeding, peer support, randomised controlled trial

## Abstract

The UK has low breastfeeding rates, with socioeconomic disparities. The Assets‐based feeding help Before and After birth (ABA) intervention was designed to be inclusive and improve infant feeding behaviours. ABA is underpinned by the behaviour change wheel and offers an assets‐based approach focusing on positive capabilities of individuals and communities, including use of a Genogram. This study aimed to investigate feasibility of intervention delivery within a randomised controlled trial (RCT). Nulliparous women ≥16 years, (*n* = 103) from two English sites were recruited and randomised to either intervention or usual care. The intervention – delivered through face‐to‐face, telephone and text message by trained Infant Feeding Helpers (IFHs) – ran from 30‐weeks' gestation until 5‐months postnatal. Outcomes included recruitment rates and follow‐up at 3‐days, 8‐weeks and 6‐months postnatal, with collection of future full trial outcomes via questionnaires. A mixed‐methods process evaluation included qualitative interviews with 30 women, 13 IFHs and 17 maternity providers; IFH contact logs; and fidelity checking of antenatal contact recordings. This study successfully recruited women, including teenagers, from socioeconomically disadvantaged areas; postnatal follow‐up rates were 68.0%, 85.4% and 80.6% at 3‐days, 8‐weeks and 6‐months respectively. Breastfeeding at 8‐weeks was obtained for 95.1% using routine data for non‐responders. It was possible to recruit and train peer supporters to deliver the intervention with adequate fidelity. The ABA intervention was acceptable to women, IFHs and maternity services. There was minimal contamination and no evidence of intervention‐related harm. In conclusion, the intervention is feasible to deliver within an RCT, and a definitive trial required.

**Key messages**

- The ABA intervention was acceptable to women, Infant Feeding Helpers and maternity providers and feasible to deliver within a randomised controlled trial with adequate fidelity. The intervention should be tested for effectiveness and cost‐effectiveness in a definitive randomised controlled trial.
- Researchers approaching women in community antenatal clinics successfully recruited teenagers and women living in socioeconomically disadvantaged areas. Introducing the research as an ‘infant feeding’ study enabled recruitment of women intending to formula feed.
- Infant Feeding Helpers were able to offer a woman‐centred approach using assets‐based conversations that included behaviour change techniques.
- There was notable difference between the two study sites in terms of level of contact between Infant Feeding Helpers and women. Context‐specific factors are important in explaining some of this difference.

## 1 INTRODUCTION

Despite the benefits of breastfeeding for infants and mothers (Victora et al., 2016), the UK experiences a high drop off in breastfeeding in the two weeks following birth, very low proportions of babies exclusively breastfed to four or six months, and marked socio‐economic inequalities in breastfeeding (McAndrew et al., 2012).

There is strong systematic review evidence that providing additional support to women who want to breastfeed increases breastfeeding duration (McFadden et al., 2017). In the UK, provision of breastfeeding peer support is recommended among disadvantaged populations (Department of Health and Department for Children Schools and Families, 2009; National Institute for Health and Care Excellence, 2008); but the coverage is variable (Grant et al., 2018). However, UK breastfeeding peer support trials have not demonstrated efficacy, possibly due to insufficiently intensive interventions, postnatal contact not commencing until after the crucial first 48 hours post hospital discharge, and contact being reactive rather than proactive (Jolly et al., 2012).

Evidence suggests more intensive contact (Jolly et al., Jolly et al., 2012b, McFadden et al., 2017) and early contact postnatally (Hoddinott, Craig, Maclennan, Boyers, & Vale, 2012a; Ingram, MacArthur, Khan, Deeks, & Jolly, 2010) are important characteristics of effective breastfeeding support. Proactive contact was found to be effective when delivered by peer supporters (Dennis, Hodnett, Gallop, & Chalmers, 2002; Forster et al., 2019), and promising in a feasibility study of an infant feeding team (Hoddinott, Craig, Britten, & McInnes, 2012c; Hoddinott, Craig, Maclennan, et al., 2012a). Woman‐centred rather than breastfeeding‐focussed support may improve acceptability to women (Hoddinott, Craig, Britten, & McInnes, 2012c; Trickey & Newburn, 2014). In cultures such as the UK, where mixed feeding is common, inclusion of help with formula feeding in peer support may be important to reduce the risk of alienating women and improve reach and retention of any intervention (Thomson, Ebisch‐Burton and Flaking, 2015; Trickey & Newburn, 2014). The ABA intervention combined all these components within an assets‐based approach (Aradon, 2007; McLean, 2011) underpinned by behaviour change theory which considered the capability, opportunity and motivation for infant feeding mode in line with the COM‐B model of the Behaviour Change Wheel framework (Michie, Atkins, & West, 2014). Assets based approaches and behaviour change theory are complimentary. The assets‐based approach informed the style and principles of intervention delivery, and the Behaviour Change Wheel informed intervention content in the form of specific Behaviour Change Techniques (BCTs) based on behavioural theory.

Assets‐based approaches to public health concentrate on positive capabilities, rather than deficits or needs, and aim to understand and maximise the strengths of individual and community resources (Aradon, 2007; McLean, 2011). Breastfeeding assets include resources that are both intrinsic (especially self‐efficacy related to feeding and the willingness to ask for and accept help) and extrinsic (including social support from a partner, friends and family; social networks of women who have breastfed and community assets such as breastfeeding groups and peer supporters).

The overall aim of this study was to determine the feasibility of delivering the ABA intervention within a randomised controlled trial (RCT). The trial protocol is published (Jolly et al., 2018) with progression criteria for a full trial. This paper reports the feasibility study findings relating to the following objectives:

- To determine intervention uptake and engagement; fidelity of intervention delivery, contamination, and acceptability to the mothers, infant feeding helpers (IFHs) and other maternity services providers;
- To determine the feasibility of the RCT processes: recruit women from socio‐economically disadvantaged populations, including teenagers and those living in areas of low breastfeeding prevalence; retain women in the study; determine the variability of the primary outcome for a future RCT; explore women's perspectives on trial processes; describe feeding support received by the ‘usual care group’; and to determine the feasibility of data collection to assess the future cost‐effectiveness of the intervention.
- To explore delivery by paid and volunteer feeding helpers, particularly acceptability and fidelity of the intervention.

## 2 METHODS

### 2.1 Study design

An individually randomised controlled feasibility trial was undertaken with women randomised on a 1:1 ratio to either the ABA intervention or the comparator (usual care).

### 2.2 Setting and participants

The study was undertaken at two distinct geographical sites in England, selected because they had contrasting volunteer and paid peer support services operating, as well as relatively high levels of socioeconomic disadvantage and low rates of breastfeeding initiation and continuation. Women were eligible if they were aged 16 years or older and pregnant with their first child. Potential participants were provided with study information by community midwives at around 25–28 weeks gestation and subsequently approached by a researcher at antenatal clinics to gain informed consent and complete a short baseline questionnaire including questions on demographics, feeding intentions and wellbeing. We aimed to recruit at least 100 women to the study (50 per site).

### 2.3 Randomisation

At Site A, an independent statistician devised a block randomisation list stratified by age group (<25 or ≥ 25 years), inaccessible to the recruiting researcher. Once a woman had given consent and completed the baseline questionnaire, the researcher telephoned the randomisation line.

At Site B, a different process was required to make sure that the number of women randomised to the intervention arm matched volunteer peer supporter availability and capacity in each sub‐locality. A clinical trials unit devised a database to randomise (simultaneously) blocks of women from each sub‐locality, following recruitment. In the case of there being an odd number of women, allocation favoured the intervention. An independent researcher performed the randomisation.

### 2.4 Intervention

Women allocated to the intervention arm were assigned an IFH, an existing peer supporter who had received a full‐day training in delivery of the ABA intervention. HT led on the development of training materials and training delivery with input from Dr Kirsty Darwent (Programme Director, Family Therapy Training Network Ltd). The training aimed to (1) promote competence and confidence in intervention delivery, and (2) facilitate understanding of the study to improve fidelity of intervention delivery. The involved simulations and role‐play of contact with women alongside group‐based learning activities. Full details of the intervention and training are available (Jolly et al., 2018). At Site A, the intervention was delivered by a paid peer support service, whereas at Site B the peer support service was provided by volunteers.

The intervention offered proactive, woman‐centred support using an assets‐based approach and incorporating behaviour change techniques (BCTs). Woman‐centred support recognises each woman as an individual and supports her to make her own decision about how she feeds her baby. Core BCTs for the antenatal part of the intervention were ‘social support’ and ‘restructuring the social environment’. Based on the COM‐B model, the core BCTs are targeting Motivation (reflective) and Opportunity (social). The assets‐based and women‐centred approach also targeted Motivation (reflective) as well as Capability (psychological). Social support could be targeted by the IFH encouraging a woman to draw on family and friends for support or by providing direct support; restructuring the social environment could be targeted by encouraging a woman to attend a postnatal group. More information on intervention development including the full list of BCTs can be found in the protocol paper (Jolly et al., 2018).

The intervention commenced between 30 and 32 weeks gestation when IFHs contacted women to offer a face‐to‐face meeting to discuss infant feeding. This antenatal meeting took place either at the woman's home (Site A only) or at a mutually convenient location, such as a café or Children's Centre. IFHs introduced the intervention and explored the woman's assets for infant feeding. This conversation led to co‐production of a ‘Genogram’ (family and social network diagram adapted from Darwent, McInnes, & Swanson, 2016) of support available to the woman, incorporating the wider community‐based assets for infant feeding. Women were encouraged to use this support network to engage in conversations about infant feeding before and after birth. IFHs also provided women with a specially designed leaflet detailing help available locally to support infant feeding and to develop social networks, and offered to accompany women to a local breastfeeding drop‐in session before birth.

The intervention continued with monthly telephone conversations/text messages during pregnancy, aiming to build strong rapport and encouraging the woman to let the IFH know once she had given birth, in order to commence postnatal support.

Postnatally, daily telephone/text message contact was provided for the first two weeks, decreasing in frequency from two to eight weeks, and monthly text messages were sent at 3, 4 and 5 months. Home visits (Site A) or meetings at convenient locations were arranged. Women were able to stop contacts at any point. If a woman ceased breastfeeding, the IFH established that the woman was confident in formula feeding and support discontinued.

### 2.5 Comparator

Women assigned to the comparator arm received the usual care provided for infant feeding within their locality. This did not include any proactive support from peer supporters either antenatally or postnatally. Women were given a leaflet detailing usual care services to support infant feeding.

### 2.6 Assessment of feasibility of delivery and acceptability of the intervention

A process evaluation was undertaken to assess (1) feasibility of intervention delivery, including protocol fidelity, and (2) intervention acceptability to women, IFHs and maternity services.

Process measures included: (1) Programme reach, assessed by recruitment and retention rates and demographic characteristics of participants, (2) Fidelity of delivery and use of assets for feeding support, assessed by content analysis of recorded antenatal meetings, IFH activity logs and qualitative interviews with women and IFHs, (3) Views of women, IFHs and representatives from local maternity services on intervention acceptability, assessed through qualitative interviews and (4) Presence of social desirability bias, assessed through comparison of IFH activity logs, qualitative interviews with women and IFHs, and feeding method reported at 8‐weeks.

### 2.7 Qualitative methods/analysis

Thirty women (21 intervention: Site A = 10, Site B = 11) were interviewed postnatally at home mainly after 8‐week follow‐up, purposively sampled for diversity (including teenagers (*n* = 3), women in areas of socioeconomic disadvantage, women with different feeding experiences (gauged from 8‐week questionnaire), and women with different levels of intervention engagement) to explore their experiences of the intervention. Control participants were asked about experiences of ‘usual care’. Possible cases of contamination between intervention and control groups were explored with all women.

Focus groups were held after completion of the intervention with IFHs at each site (*n* = 9) (followed by one‐to‐one telephone interviews for those unable to attend (*n* = 4)). They investigated intervention acceptability, satisfaction with the training, experiences of intervention delivery and any perceived barriers or facilitators to effective delivery.

Focus groups and interviews were also undertaken with maternity care providers (*n* = 17), including community midwives and Infant Feeding staff. These explored perceptions of the intervention, any impact of intervention provision on existing services, and any possible cases of contamination.

Interviews and focus groups were carried out by researchers from psychology, public health and midwifery backgrounds and with training in qualitative research methods (JC, DJ, JI and GT). JI and GT also have experience of research/evaluation into breastfeeding peer support. JC and DJ who carried out the women's interviews and with JI the maternity services interviews/focus groups had met the women and some of the community midwives previously at recruitment. GT who led on the IFH focus groups/interviews had no previous contact with IFHs.

Discussions with women lasted between 45 and 90 minutes. IFH focus groups lasted ~90 minutes and IFH interviews were about 30–60 minutes. Maternity services focus groups and interviews lasted 30–60 minutes. Reflective notes were made after each interview. All interviews were voice‐recorded and transcribed verbatim. Transcripts were checked for accuracy and anonymised.

We undertook thematic analysis (Braun & Clarke, 2006) of the qualitative data using NVivo 11 (QSR International Pty Ltd. Version 11, 2015). First, three researchers (JC, DJ and GT) listened to the recordings and read/re‐read the transcripts of four participant interviews (one intervention and one usual care from each site) before independently conducting line‐by‐line inductive coding. Codes were discussed and developed into an initial coding framework of themes and sub‐themes. JC and DJ then coded the remaining transcripts using the coding framework, which was iteratively refined to accommodate new themes. There were frequent discussions between the three researchers during the development of the coding framework and before the final coding framework was agreed by the wider team (JC, DJ, GT, JI, SD, KJ).

For each of the women's interviews, BCTs delivered by IFHs were coded as standalone themes, based on reports of the IFH behaviour, regardless of participant response. BCTs delivered by people other than the IFHs (e.g. midwives) were not coded in this analysis.

### 2.8 Assessment of fidelity

IFHs were asked to audio‐record antenatal visits. Recordings were analysed against fidelity criteria and a checklist of core/non‐core BCTs. Additionally, qualitative interviews with women were checked for reports of BCTs and woman‐centredness.

IFHs were asked to log each contact with women, recording mode of contact and response received.

### 2.9 Outcomes for a future trial

Data were collected on breastfeeding, health‐related and economic outcomes to explore feasibility of data collection for a future definitive trial. These included the proposed primary outcome for a definitive trial – any breastfeeding at 8‐weeks – and a number of secondary outcomes: breastfeeding initiation; exclusive breastfeeding at 6–8 weeks; any/exclusive breastfeeding at 6‐months; duration of any breastfeeding (if ceased); maternal wellbeing (Warwick‐Edinburgh Mental Well‐being Scale) (Tennant et al., 2007) at 8‐weeks and 6‐months, maternal satisfaction with feeding experience and support (single‐item scale from Hoddinott, Craig, Maclennan, et al., 2012a), use of health and feeding support services and receipt of benefits at 8‐weeks.

Outcome data were collected at three time‐points. At 2–3 days postnatally, participants were sent a text message asking them to respond with their feeding method since birth (formula milk, breastmilk or both). At 8‐weeks and 6‐months postnatally, women were sent a questionnaire to complete and return by post (or by telephone if preferred). Women were sent a £25 shopping voucher following return of the 6‐month questionnaire. Routinely collected health visitor data were accessed for missing 8‐week feeding outcomes.

### 2.10 SAMPLE SIZE

We calculated that a sample size of 100 women would allow a reasonable level of precision in estimation of feasibility outcomes, enabling bounds for 95% confidence intervals (CIs) for recruitment, follow‐up and questionnaire completion to be within 15% of the estimate calculated using an estimate of 50% for all outcomes.

### 2.11 STATISTICAL ANALYSIS

We used STATA 15 (Texas, USA) for statistical analysis. Descriptive statistics were used to outline participant characteristics by site and randomisation allocation.

To measure trial feasibility, we reported recruitment and follow‐up rates (with 95% binomial exact CIs) and data completeness. We described number and method of IFH contacts with women in the intervention and control arms to assess level of intervention delivery and any contamination in the control group.

Although this study was not powered to ascertain differences between intervention and control arms, we calculated percentages (with 95% CIs) for breastfeeding and health‐related outcome measures. The variability in the primary outcome between IFHs was assessed by calculating the intra‐cluster correlation coefficient (ICC) using a null linear model with a random effect for IFH. These data will inform sample size calculation for a future definitive trial. We describe women's characteristics by allocation group and present summaries for each outcome measure. Primary analysis was by modified intention to treat, which included all randomly assigned patients with available data on the primary endpoint (self‐report or routinely collected).

### 2.12 ETHICAL CONSIDERATIONS

Ethical approval was received in November 2016 from South West – Cornwall and Plymouth Research Ethics Committee (16/SW/0336). The study was registered with the International Standard Randomised Controlled Trial Register (ISRCTN14760978).

## 3 RESULTS

### 3.1 Participant recruitment and follow‐up

**Figure 1:**
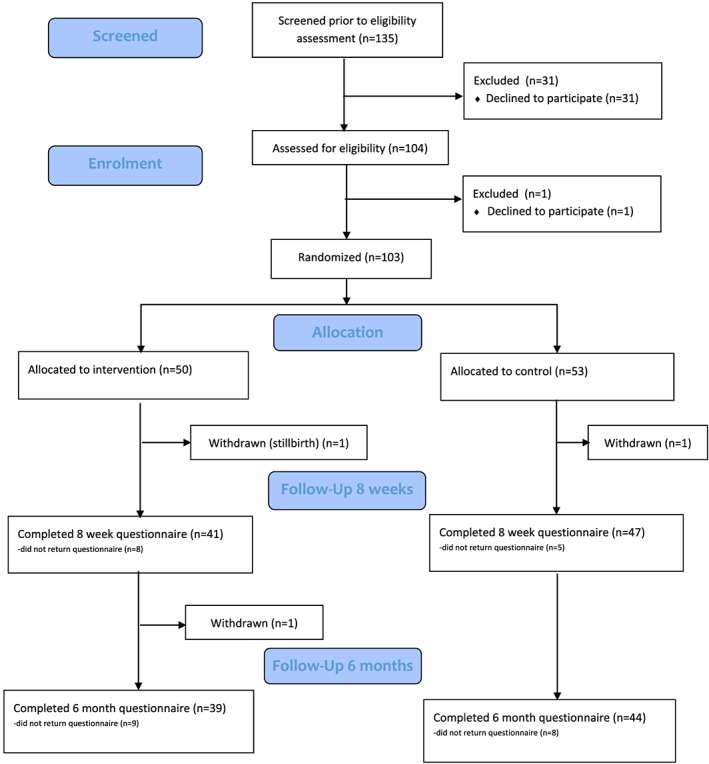
Flow diagram to show participant flow through the ABA study

Of 135 eligible women invited to participate, 103 (76.3%, 95% CIs: 68.2–83.2%) consented and were randomly assigned to the intervention (*n* = 50) and usual care (*n* = 53) groups (Figure 1). Recruitment took place February–May 2017 at Site A and April–August 2017 at Site B. Recruitment finished when at least 50 women had been recruited from each site.

Participant characteristics are shown in Table 1. The sample included nine teenagers (8.7%), and 38 women (37.3%) from the two most deprived Index of Multiple Deprivation quintiles. Fourteen women (13.9%) intended to feed their baby either ‘formula milk only’ or ‘mainly formula’.

**Table 1:** Demographic and delivery characteristics of participants

	**Site A**			**Site B**			**Overall**		
**Characteristic**	**Intervention** *N* = 25	**Usual care** *N* = 28	**All** N = 53	**Intervention** N = 25	**Usual care** N = 25	**All** N = 50	**Intervention** N = 50	**Usual care** N = 53	**All** N = 103
**Age at baseline (years), mean (SD)**	27.9 (5.2)	27.7 (5.9)	27.8 (5.5)	29.2 (20.5)	29.3 (5.6)	29.3 (5.4)	28.6 (5.2)	28.5 (5.8)	28.5 (5.5)
**Age range, minimum‐maximum (years)**	17.7–37.7	17.9–39.0	17.7–39.0	20.5–43.0	17.9–42.9	17.9–43.0	17.7–43.0	17.9–42.9	17.7–43.0
Missing, n (%)	0 (0)	1 (3.6)	1 (1.9)	0 (0)	0 (0)	0 (0)	0 (0)	1 (1.9)	1 (1.0)
**Ethnicity, n (%)**									
White British	21 (84.0)	22 (81.5)	43 (82.7)	22 (88.0)	23 (92.0)	45 (90.0)	43 (86.0)	45 (86.5)	88 (86.3)
White other	1 (4.0)	3 (11.1)	4 (7.7)	2 (8.0)	1 (4.0)	3 (6.0)	3 (6.0)	4 (7.7)	7 (6.9)
Asian	0 (0)	0 (0)	0 (0)	0 (0)	1 (4.0)	1 (2.0)	0 (0)	1 (1.9)	1 (1.0)
Black African	0 (0)	1 (3.7)	1 (1.9)	0 (0)	0 (0)	0 (0)	0 (0)	1 (1.9)	1 (1.0)
Black Caribbean	1 (4.0)	0 (0)	1 (1.9)	0 (0)	0 (0)	0 (0)	1 (2.0)	1 (1.9)	1 (1.0)
Mixed	1 (4.0)	1 (3.7)	2 (3.9)	1 (4.0)	0 (0)	1 (2.0)	2 (4.0)	1 (1.9)	3 (2.9)
Other	1 (4.0)	0 (0)	1 (1.9)	0 (0)	0 (0)	0 (0)	1 (2.0)	0 (0)	1 (1.0)
Missing	0 (0)	1 (3.6)	1 (1.9)	0 (0)	0 (0)	0 (0)	0 (0)	1 (1.9)	1 (1.0)
**Employment status, n (%)**									
In paid work	18 (72.0)	25 (92.6)	43 (82.7)	22 (88.0)	25 (100)	47 (94.0)	40 (80.0)	50 (96.2)	90 (88.2)
Unemployed	6 (24.0)	1 (3.7)	7 (13.5)	2 (8.0)	0 (0)	2 (4.0)	8 (16.0)	1 (1.9)	9 (8.8)
Full‐time education or training	0 (0)	1 (3.7)	1 (1.9)	1 (4.0)	0 (0)	1 (2.0)	1 (2.0)	1 (2.0)	2 (2.0)
Missing	0 (0)	1 (3.6)	1 (1.9)	0 (0)	0 (0)	0 (0)	0 (0)	1 (1.9)	1 (1.0)
**Highest level of qualification, n (%)**									
No formal qualification	1 (4.0)	0 (0)	1 (1.9)	0 (0)	0 (0)	0 (0)	1 (2.0)	0 (0)	1 (1.0)
GCSE or equivalent	6 (24.0)	5 (18.5)	11 (21.2)	6 (24.0)	5 (20.0)	11 (22.0)	12 (24.0)	10 (19.2)	22 (21.6)
A/AS‐level or equivalent	8 (32.0)	6 (22.2)	14 (26.9)	12 (48.0)	7 (28.0)	19 (38.0)	20 (40.0)	13 (25.0)	33 (32.4)
Degree level or higher	10 (40.0)	16 (59.3)	26 (50.0)	7 (28.0)	13 (52.0)	20 (40.0)	17 (34.0)	29 (55.8)	46 (45.1)
Missing	0 (0)	1 (3.6)	1 (1.9)	0 (0)	0 (0)	0 (0)	0 (0)	1 (1.9)	1 (1.0)
**Relationship status, n (%)**									
Married/registered civil partnership	9 (36.0)	12 (46.2)	21 (41.2)	13 (52.0)	14 (56.0)	27 (54.0)	22 (44.0)	26 (51.0)	48 (47.5)
Living together	9 (36.0)	11 (42.3)	20 (39.2)	9 (36.0)	9 (36.0)	18 (36.0)	18 (36.0)	20 (39.2)	38 (37.6)
Single	7 (28.0)	3 (11.5)	10 (19.6)	3 (12.0)	2 (8.0)	5 (10.0)	10 (20.0)	5 (9.8)	15 (14.9)
Missing	0 (0)	2 (7.1)	2 (3.8)	0 (0)	0 (0)	0 (0)	0 (0)	2 (3.8)	2 (1.9)
**Index of multiple deprivation quintile, n (%)**									
1 (most deprived)	13 (52.0)	11 (40.7)	24 (46.2)	1 (4.0)	0 (0)	1 (2.0)	14 (28.0)	11 (21.2)	25 (24.5)
2	3 (12.0)	6 (22.2)	9 (17.3)	2 (8.0)	2 (8.0)	4 (8.0)	5 (10.0)	8 (15.4)	13 (12.8)
3	1 (4.0)	7 (25.9)	8 (15.4)	8 (32.0)	3 (12.0)	11 (22.0)	9 (18.0)	10 (19.2)	19 (18.6)
4	7 (28.0)	3 (11.1)	10 (19.2)	6 (24.0)	11 (44.0)	17 (34.0)	13 (26.0)	14 (26.9)	27 (26.5)
5 (least deprived)	1 (4.0)	0 (0)	1 (1.9)	8 (32.0)	9 (36.0)	17 (34.0)	9 (18.0)	9 (17.3)	18 (17.7)
**Maternal wellbeing (WEMWBS), mean (SD)**	54.1 (9.8)	55.0 (9.2)	54.6 (9.4)	53.4 (6.2)	53.7 (8.4)	53.6 (7.3)	53.7 (8.1)	54.4 (8.7)	54.1 (8.4)
Missing, n (%)	0 (0)	1 (3.6)	1 (1.9)	0 (0)	0 (0)	0 (0)	0 (0)	1 (1.9)	1 (1.0)
**Intention to feed, n (%)**									
Breastmilk only	10 (40.0)	9 (33.3)	19 (36.5)	7 (28.0)	9 (37.5)	16 (32.7)	17 (34.0)	18 (35.3)	35 (34.7)
Mainly breastmilk	7 (28.0)	7 (25.9)	14 (26.9)	10 (40.0)	6 (25.0)	16 (32.7)	17 (34.0)	13 (25.5)	30 (29.7)
Half and half	4 (16.0)	6 (22.2)	10 (19.2)	6 (24.0)	6 (25.0)	12 (24.5)	10 (20.0)	12 (23.5)	22 (21.8)
Mainly formula	2 (8.0)	2 (7.4)	4 (7.7)	1 (4.0)	0 (0)	1 (2.0)	3 (6.0)	2 (3.9)	5 (5.0)
Formula milk only	2 (8.0)	3 (11.1)	5 (9.6)	1 (4.0)	3 (12.5)	4 (8.2)	3 (6.0)	6 (11.8)	9 (8.9)
Missing	0 (0)	1 (3.6)	1 (1.9)	0 (0)	1 (4.0)	1 (2.0)	0 (0)	2 (3.8)	2 (1.9)
**How participant was fed as a baby, n (%)**									
Breastfed entirely	7 (28.0)	8 (29.6)	15 (28.9)	9 (36.0)	12 (48.0)	21 (42.0)	16 (32.0)	20 (38.5)	36 (35.3)
Formula fed entirely	8 (32.0)	13 (48.2)	21 (40.4)	5 (20.0)	3 (12.0)	8 (16.0)	13 (26.0)	16 (30.8)	29 (28.4)
Mixed feeding	10 (40.0)	5 (18.5)	15 (28.9)	7 (28.0)	6 (24.0)	13 (26.0)	17 (34.0)	11 (21.2)	28 (27.5)
Do not know	0	1 (3.7)	1 (1.9)	4 (16.0)	4 (16.0)	8 (16.0)	4 (8.0)	5 (9.6)	9 (8.8)
Missing	0 (0)	1 (3.6)	1 (1.9)	0 (0)	0 (0)	0 (0)	0 (0)	1 (1.9)	1 (1.0)
**Knows anyone who has breastfed their baby, n (%)**	22 (88.0)	25 (92.6)	47 (90.4)	21 (84.0)	25 (100)	46 (92.0)	43 (86.0)	50 (96.2)	93 (91.2)
Missing	0 (0)	1 (3.6)	1 (1.9)	0 (0)	0 (0)	0 (0)	0 (0)	1 (1.9)	1 (1.0)
**Gestational age at birth (weeks), mean (SD)**	39.0 (2.3)	40.1 (1.2)	39.6 (1.9)	39.7 (1.7)	39.3 (1.8)	39.5 (1.8)	39.4 (2.0)	39.7 (1.6)	39.5 (1.8)
Missing	1 (2.0)	1 (3.6)	2 (3.8)	0 (0)	0 (0)	0 (0)	1 (2.0)	1 (1.9)	2 (1.9)
**Premature baby, n (%)**	5 (20.8)	0 (0)	5 (9.8)	2 (8.0)	2 (8.0)	4 (8.0)	7 (14.3)	2 (3.9)	9 (8.9)
Missing	1 (2.0)	1 (3.6)	2 (3.8)	0 (0)	0 (0)	0 (0)	1 (2.0)	1 (1.9)	2 (1.9)
**Mode of delivery, n (%)**									
Vaginal birth	5 (26.3)	10 (50.0)	15 (38.5)	10 (47.6)	12 (50.0)	22 (48.9)	15 (37.5)	22 (50.0)	37 (44.1)
C‐section (planned)	1 (5.3)	1 (5.0)	2 (5.1)	2 (9.5)	2 (8.3)	4 (8.9)	3 (7.5)	3 (6.8)	6 (7.1)
C‐section (emergency)	4 (21.1)	4 (20.0)	8 (20.5)	4 (19.1)	6 (25.0)	10 (22.2)	8 (20.0)	10 (22.7)	18 (21.4)
Forceps, ventouse, vacuum delivery	9 (47.4)	5 (25.0)	14 (35.9)	5 (23.8)	4 (16.7)	9 (20.0)	14 (35.0)	9 (20.5)	23 (27.4)
Missing	6 (24.0)	8 (28.6)	14 (26.4)	4 (16.0)	1 (4.0)	5 (10.0)	10 (20.0)	9 (17.0)	19 (18.4)
**Duration of mother hospital stay, n (%)**									
<24 hrs	3 (15.8)	6 (30.0)	9 (23.1)	5 (23.8)	4 (16.7)	9 (20.0)	8 (20.0)	10 (22.7)	18 (21.4)
24‐48 hrs	11 (57.9)	6 (30.0)	17 (43.6)	7 (33.3)	6 (25.0)	13 (28.9)	18 (45.0)	12 (27.3)	30 (35.7)
>48 hrs	5 (26.3)	7 (35.0)	12 (30.8)	9 (42.9)	14 (58.3)	23 (51.1)	14 (35.0)	21 (47.7)	35 (41.7)
Home birth	0	1 (5.0)	1 (2.6)	0 (0)	0 (0)	0 (0)	0 (0)	1 (2.3)	1 (1.2)
Missing	6 (24.0)	8 (28.6)	14 (26.4)	4 (16.0)	1 (4.0)	5 (10.0)	10 (20.0)	9 (17.0)	19 (18.4)
**Baby admitted to neonatal unit, n (%)**	4 (21.1)	2 (10.0)	6 (15.4)	3 (14.3)	2 (8.3)	5 (11.1)	7 (17.5)	4 (9.1)	11 (13.1)
Missing	6 (24.0)	8 (28.6)	14 (26.4)	4 (16.0)	1 (4.0)	5 (10.0)	10 (20.0)	9 (17.0)	19 (18.4)

WEMWBS = Warwick‐Edinburgh Mental Wellbeing Scale^97^ (score ranging from 14 to 70; 70 indicates highest level of mental wellbeing)

Late birth notifications (median age of baby when IFH notified of birth = 3 days, IQR 0, 30) resulted in delays sending out the postnatal text to collect feeding status at 2–3 days. We were able to send a postnatal text to 84/103 (81.6%) women within 10 days of birth and received responses from 70 (68%, 95% CI: 58.0–76.8%) women.

Follow up questionnaires were returned by 88 (85.4%, 95% CI: 77.1–91.6%) and 83 (80.6%, 95% CI: 71.6–87.7%) women at 8‐weeks and 6‐months respectively. We accessed routine health visitor data for an additional 10 participants who did not return their 8‐week questionnaire, meaning we had available data on ‘any breastfeeding at 8‐weeks’ for 95.1% (95% CI: 89.0–98.4%) of women.

Comparison of demographic characteristics of responders and non‐responders revealed that non‐responders were: younger; more were White British, single and breastfeeding at 8‐weeks; and, fewer were employed, educated to degree level, and reported intention to breastfeed at baseline [Table S1].

Two women withdrew from the study (one immediately after randomisation – no reason given, and one between the two follow‐ups – no longer wanted to participate). One woman suffered a stillbirth and was withdrawn by the study team.

### 3.2 *Women's and maternity services providers' views on recruitment and randomisation processe*s

All the women interviewed found the recruitment processes and timing acceptable but would not have wanted to be approached before the 20‐week scan.

I did not really want to acknowledge until the 20‐week scan, … 12‐weeks … I do not think I was even thinking about post birth. (Participant 16 – Intervention, Site B).

While there were variations as to when women received the study leaflet ‐ some received the leaflet early, others received it on the day of recruitment ‐ this did not affect women's willingness to be involved, although there was a preference for receiving the invitation earlier.

I guess if the midwife of the previous appointment said there's a feeding study going on, this is the leaflet about what they are doing, they are going to be here next time and they might want to have a chat with you, then I suppose that could have given me a bit more time to have a think about it. But I wasn't really thinking I wish I had more time to think about it or anything like that. (Participant 17 – Usual care, Site B).

Women provided diverse responses regarding midwifery staff involvement in study recruitment. Some felt it was more important to discuss the purpose and practicalities with the researcher, whilst others felt that midwifery endorsement helped to authenticate the study.

*I probably would not have done anything if it was just you [researcher] if I was honest, it was because my midwife said… this is a research would you want to take part? … it was nice to have that confirmation that it is an actual study going on*. *(Participant 28 – Usual care, Site B)*.

Overall, women across both study arms found the randomisation process to be acceptable. Women wanted to be part of a study, which may or may not have direct personal benefits, but might make a difference to others.

The study for us I just wanted to be part of it in regards to if it helps somebody else, if it helps us in the future, but if it helps somebody then it's worth being part of. (Participant 2 – Intervention, Site A).

The midwives did not experience any particular difficulties in giving women the leaflets or introducing the study. They valued the researcher's presence and their knowledge and time to explain the study. None of the midwives interviewed experienced any problems in women not wanting to participate. This they believed could be attributed in part to their personal introductions, such as *‘we've got a study’*, thereby demonstrating their endorsement. Some professionals also considered women were willing to participate due to the general approach being *‘infant feeding rather than just breastfeeding’*.

#### 3.2.1 Infant feeding helper recruitment and training

We were able to recruit a sufficient number of existing peer supporters to the ABA IFH role, with 13 out of a possible 16 peer supporters agreeing to be involved.

Although, overall, IFHs reported the ABA training to be acceptable, IFHs at Site B were generally more positive about it than IFHs at Site A:

The role play was really useful … and doing the genogram was really useful, having a bit of the formula section was really useful because … it's not something I know a lot about, but that was helpful. (IFH – Site B, Focus Group).

IFHs at Site A felt that the training offered little new to them and were uncertain about the perceived ‘*prescriptive*’ nature of the intervention:

I thought the whole conversation thing [role play] was a little bit patronising, because it's what we do anyway … it was a bit like we knew how to sit and talk to mums, so other than that though it was fine. (IFH – Site A, Interview).

#### 3.2.2 Intervention delivery and uptake

IFH activity logs were provided for 49/50 (98%) women. The missing log related to a woman the IFH had been unable to contact. IFHs attempted to contact all women assigned to the intervention arm to offer an antenatal visit (Table 2). In total 39/50 (78%) women completed an antenatal meeting, four (8%) could not be contacted, four (8%) gave birth prematurely before contact was established, two (4%) withdrew from the intervention and one (2%) declined. No women took up the offer to be accompanied to a breastfeeding group antenatally.

**Table 2:** Infant feeding helper reported contact with women

	Site A	Site B	Overall
Antenatal contact attempted	25/25 (100%)	25/25 (100%)	50/50 (100%)
Antenatal visit completed	17/25 (68%)	22/25 (88%)	39/50 (78%)
Postnatal contact attempted^a^	24/24 (100%)	22/25 (88%)	46/49 (93.9%)
Postnatal support provided	18/24 (75%)	22/25 (88%)	40/49 (81.6%)
Contact attempted by infant feeding helper within 48 hours of birth	6/24 (25%)	18/25 (72%)	24/49 (49%)
Number of days contact made/attempted by IFH in 14 days postnatal, median (IQR)	*N* = 24 2.5 (1.5,3)	N = 25 8 (4,14)	*N* = 49 4 (2,8)
Number of days two‐way contact established in 14 days postnatal, median (IQR)	N = 24 1 (0,2)	N = 25 7 (3,13)	N = 49 2 (1,7)
Number (%) of days contact made/attempted by IFH in 14 days postnatal (denominator women who were known to be breastfeeding (from 8wQ) that IFH had been informed about birth)	Eligible days for support=162^b^ 29 (17.9%)	Eligible days for support=235^c^ 198 (84.3%)	Eligible days for support = 397 227 (57.2%)
Number of two‐way contact days from 2 to 8 weeks postnatal, median (IQR)	N = 24 1 (0,2)	N = 25 4 (1,7)	N = 49 2 (0,4)
Support provided 8‐weeks to 5‐months	9/24 (37.5%)	15/25 (60%)	24/49 (49.0%)

a one woman suffered stillbirth and was withdrawn from the study.

b excludes stillbirth (*n* = 1), no 8‐week questionnaire data (*n* = 4), no IFH notes available (n = 1).

c excludes declined support (*n* = 2), out of the country (n = 1).

Postnatally, IFHs attempted to contact 46/49 (93.9%) women to offer support, with 24/49 (49.0%) contacted within 48 hours of birth; one woman had a stillbirth so is omitted from the denominator. Forty women (81.6%) received postnatal support, five (10%) could not be contacted and one woman declined support. At Site A, IFHs reported home visits to 7/24 (29.2%) women postnatally. In the first two postnatal weeks the IFH sent a text/call on a median of 4 days (IQR 2,8). The median number of days in which two‐way contact between IFH and a woman occurred was 2 days (IQR 1,7) in the first 2‐weeks postnatally, and 2 days (IQR 0,4) from 2 to 8‐weeks postnatally. For women known to be breastfeeding in the first two weeks postnatally, IFHs made or attempted contact on 57.2% of possible days after they had been notified of birth. Between 8‐weeks and 5‐months postnatally, 24/49 women (49.0%) received some support. There was notable variation between sites, with Site B IFHs maintaining a considerably higher level of contact. Many women reported that they preferred to text.

… text message was better because at that point I was always feeding him, so it was quite difficult to get the phone, so with the text it was more easy because I just answer when I could and she [IFH] the same. (Participant 27, Site B).

#### 3.2.3 Intervention fidelity

Fidelity checking was undertaken on 18 recordings of antenatal meetings (two Site A; 16 Site B). Results suggest that woman‐centred assets‐based conversations, including BCTs, can be delivered by IFHs. Analysis of qualitative interviews with women showed IFHs were able to offer a woman‐centred approach. There was evidence of delivery of the core BCTs ‘social support’ and ‘restructuring the social environment’ (reported in 18/21 and 20/21 interviews with intervention women respectively). IFHs completed a genogram with 38 of the 39 women who took part in an antenatal meeting.

#### 3.2.4 Intervention acceptability

Qualitative analysis showed the intervention was acceptable to women, IFHs and maternity services at both sites.

Women valued the opportunity of support from someone with similar experiences and learning about what was available.

I think just having that additional person to talk to makes you feel less alone … … so it puts you at ease really about how you can actually do it. I think that's essentially what you want, you want someone to have the same experiences as you, you want someone to be like no it's fine, you are okay. (Participant 2 Site A).

Overall IFHs appreciated the chance to meet women antenatally, to continue contact for several months, and offer woman‐centred support.

When you first started meeting antenatally, you were excited about it, and planning where to meet, and meeting these women. We were so amazed by the diversity of the women and that was really powerful, and how different they were to women we were meeting in our ordinary groups …, they were really different. (IFH manager Site B focus group).

The antenatal meetings between women and their IFHs were described as relaxed discussions with an opportunity to have a ‘chat’ about infant feeding whatever their preference.

It sometimes opens up that conversation .., it might be easier this way, so definitely having that information at least we know then and we do not look like we are just the breastfeeding police kind of thing, we can speak to them about what they want to do as well. So, it's good I suppose. (IFH3 Site A focus group).

Women provided mostly positive views about the mapping exercise (genogram).

*She did a really useful thing actually, which was we did a map of people in my life that I could ask any help for feeding advice and things like that … and just it just made me rethink and evaluate how much I appreciate having some family closer by*. (Participant 23 ‐ Site B).

Postnatally women appreciated the proactive contacts from their helpers and valued the range of methods of contact with the IFH, whether by phone, text or in person. With some of the women identifying the importance of the support on their infant feeding experiences.

I genuinely believe if it wasn't for the study and for [IFH] and introducing me to the breast friends' group, I do not think I would have got this far and certainly not breastfeeding exclusively. (Participant 19 Site B).

Midwives also reported on the complementary role of the intervention with usual care.

I think it would help us as well knowing that actually they are being supported that if we have not got that time necessarily that they are still being supported. (Maternity services Site A focus group).

#### 3.2.5 Usual care

Peer support services at a woman's request existed at both sites prior to and during this study. Of 42 women in the usual care arm responding to a question at 8‐weeks on use of feeding support services for advice on infant feeding, seven (16.7%) reported accessing support from an infant feeding counsellor/breastfeeding supporter and 11 (26.2%) had attended a breastfeeding group (Table S2).

#### 3.2.6 Contamination and adverse events

We identified one case of contamination. One woman at Site B reported sharing the assets leaflet with friends who were in the usual care group. The impact of this is likely to have been low as the assets leaflet represents only one component of the intervention. There were no reported adverse events related to the intervention.

#### 3.2.7 Outcomes for a definitive trial

Whilst recognising that this feasibility trial was not powered to detect differences between study arms, we found the proportion of intervention women reporting breastfeeding initiation and any breastfeeding at 8‐weeks and 6‐months was consistently higher than in the usual care group (Table 3). There was no evidence of social desirability bias. Wellbeing and satisfaction with support are reported in web‐Table 2. We demonstrated the feasibility of data collection for a future cost‐effectiveness analysis; use of feeding support services are reported in web‐Table 2.

**Table 3:** Estimates from feasibility study: Breastfeeding initiation, any and exclusive breastfeeding at 8 weeks and 6 months

	Intervention *N* = 50		Usual care N = 53		All N = 103	
	n/N	% (95%CIs)	n/N	% (95%CIs)	n/N	% (95%CIs)
Breastfeeding initiation	35/41	85.4 (70.8, 94.4)	36/47	76.6 (62.0, 87.7)	71/88	80.7 (70.9, 88.3)
Any breastfeeding at 8 weeks	23/41	56.1 (39.7, 71.5)	22/47	46.8 (32.1, 61.9)	45/88	51.1 (40.2, 61.9)
Any breastfeeding at 8 weeks (including health visitor data)^1^	24/48	50.0 (35.2, 64.8)	22/50	44.0 (30.0, 58.7)	46/98	46.9 (36.8, 57.3)
Any breastfeeding at 6 months	18/39	46.2 (30.1, 62.8)	16/44	36.4 (22.4, 52.2)	34/83	41.0 (30.3, 52.3)
Exclusive breastfeeding at 6–8 weeks (last 24 hrs)	16/41	39.0 (24.2, 55.5)	17/47	36.2 (22.7, 51.5)	33/88	37.5 (27.4, 48.5)
Exclusive breastfeeding at 6–8 weeks (since birth)	11/41	26.8 (14.2, 42.9)	12/47	25.5 (13.9, 40.3)	23/88	26.1 (17.3, 36.6)
Exclusive breastfeeding at 6 months (last 24 hrs definition)	12/39	30.8 (17.0, 47.6)	13/44	29.5 (16.8, 45.2)	25/83	30.1 (20.5, 41.2)
Exclusive breastfeeding at 6 months (no other food/drink ever definition)	3/39	7.7 (1.6, 20.9)	2/44	4.5 (0.5, 20.9)	5/83	6.0 (2.0, 13.5)

1 ICC for infant feeding helpers 0.039.

## 4 DISCUSSION

This study aimed to determine the feasibility of delivering the ABA intervention in a definitive RCT. Our results indicate that (1) we were successful in recruiting women from areas of socioeconomic disadvantage and teenagers, with adequate follow up rates; (2) it was feasible to recruit and train existing peer supporters to the new ABA role, and they were able to deliver the intervention with satisfactory fidelity, incorporating the delivery of core BCTs in line with behavioural theory and a woman‐centred approach; (3) the intervention was acceptable to women, IFHs and maternity services; and (4) there were no harms associated with the intervention, and contamination was low. To our knowledge, this is the first infant feeding study in the UK to provide woman‐centred infant feeding support to women regardless of feeding intention using an assets‐based approach.

Whilst systematic reviews of peer support report benefit (McFadden et al., 2017) UK trials of breastfeeding peer support have not been effective (Jolly et al., 2012). Many of the trials in systematic reviews are from low‐income countries, the usual care group received a lower level of support for feeding than is standard care in the UK and the interventions were often more intensive than delivered in UK trials (Jolly, Ingram, Freemantle, et al., 2012) hence the need to further explore effectiveness of feeding peer support in the UK.

An uncontrolled UK feasibility trial of a breastfeeding peer support intervention including motivational interviewing by paid peer supporters (Mam‐Kind study) (Copeland et al., 2018) and a feasibility RCT of proactive and reactive telephone support for breastfeeding women living in disadvantaged areas (FEST) study (Hoddinott, Craig, Maclennan, et al., 2012a; Hoddinott, Craig, MacLennan, Boyers, & Vale, 2012b) were both shown to be feasible and acceptable. Detailed process evaluations of these studies enable comparisons to be drawn with the ABA study.

The ABA recruitment method (researcher approaching potentially eligible women in community antenatal clinics) was more successful than the approach taken in the Mam‐Kind study where community midwives were asked to pass on women's details to the research team for recruitment. In the ABA study, our recruitment rate was 76%, versus 24% in Mam‐Kind. We also recruited a higher proportion of teenagers, women with lower educational attainment and women from ethnic minorities, possibly in part due to Mam‐Kind's exclusion of women not planning to breastfeed. The Mam‐Kind study contacted a higher proportion of women within 48‐hours of birth (73% compared to 48% in ABA). Within Mam‐Kind the midwife supervising the peer support teams encouraged hospital midwives to notify peer supporters of births. Reasonable intervention fidelity was achieved in both the Mam‐Kind and ABA studies. Mam‐Kind reported difficulties for peer supporters in moving away from information‐giving to a more collaborative approach. This resonated with the ABA study's experience of working with paid peer supporters. Some women in the Mam‐Kind study reported that cessation of support at 14‐days (with facilitated transition to a breastfeeding/community support group) felt somewhat abrupt, adding validation to the ABA approach of a longer support period and a more gradual withdrawal of support to encourage breastfeeding maintenance.

In the FEST intervention a feeding team met women face to face after birth in hospital and aimed to provide daily proactive telephone calls to breastfeeding women (*n* = 35) in the week following hospital discharge, with the option of continuing daily calls up until 14‐days; a median of eight proactive calls/woman occurred in the 14‐days following hospital discharge. In the ABA study, the number of days where two‐way contact was established between woman and IFH in the first two‐weeks postnatally varied from zero to 14, with a median of two. A lower level of two‐way contact compared to FEST was partly due to delays in birth notifications. Also, the inclusive nature of the ABA intervention meant that women who were formula feeding required less day‐to‐day support and the woman‐centred approach meant that contact frequency was negotiated. This was particularly the case at Site A, with a lower proportion of women breastfeeding.

Contextual differences between the two ABA study sites may also have contributed to the lower overall contact between IFHs and women at Site A, where there were several preterm births and more women living in socio‐economically disadvantaged and challenging circumstances, as well as uncertainty about the continuation of their peer support service. Also, at Site A paid IFHs provided support primarily within office hours, whereas at Site B volunteer IFHs were more flexible in their approach to contacting women in the evenings and at weekends.

### 4.1 Strengths and limitations

This study used robust methods including a usual care group and a comprehensive process evaluation. Delays in birth notifications were a limitation, resulting in delays in collection of postnatal feeding status data and delivery of the postnatal intervention for some women, which has been a recurring challenge in previous UK trials of peer support (Graffy, Taylor, Williams, & Eldridge, 2004; Jolly, Ingram, Freemantle, et al., 2012). All qualitative interviews were with women who returned an 8‐week questionnaire. This could have led to positive bias in the responses of interviewees, as the socio‐demographic characteristics of non‐responders at 8‐weeks were those known to be associated with lower rates of breastfeeding (McAndrew et al., 2012). For the fidelity assessment we only had two recorded antenatal meetings from Site A due to IFH concerns that recording might affect the interaction. Thus the fidelity results can only be applied with confidence to Site B. The qualitative researchers had different health related backgrounds, and some had prior experience of evaluating peer support. These qualities increased the robustness of the analysis. It is possible that IFHs may have altered the support they provided to any usual care women who they saw in a breastfeeding group (26.2% of usual care responders attended a breastfeeding group). However, the use of the genogram and initial discussion of local assets took place antenatally. No usual care women had antenatal contact with the IFHs and no contamination was reported by IFHs.

### 4.2 Recommendations for future research

We met our criteria for progression to a future trial: the intervention was acceptable to women, IFHs and health service staff; we recruited more than 75 women in 5 months; at least 5% of women recruited were teenagers; over 75% of the women in the intervention group received a contact in both the antenatal and postnatal periods and over 75% received the assets‐based contact; and data on any breastfeeding was obtained for over 80% of participants at 8 weeks and 6 months. Thus we consider that the ABA intervention was feasible to deliver within an RCT and a future definitive RCT is required to determine effectiveness and cost‐effectiveness. UK collection of routine data for feeding method at 8‐weeks by health visitors facilitates high completion for the proposed primary outcome in a full trial.

Contamination was low in this feasibility study, so we recommend an individually randomised trial with clustering by IFH accounted for in the sample size calculation for the intervention arm.

For future intervention delivery, we would need to identify localities with existing peer support services with stable commissioning and good managerial support to enable adoption of the ABA approach. Whilst a cluster RCT would reduce contamination between the intervention and comparator group, the required sample size would render such a trial not cost‐effective. We therefore recommend an individually randomised trial with any breastfeeding at 8‐weeks as the primary outcome. Such a trial would need a large sample size (>2500), and large number of sites to enhance generalisability; this would enable us to explore differences in delivery and outcomes in different contexts. Randomisation should be stratified by site to take into account different population characteristics and delivery. We recommend targeting areas with low breastfeeding rates in a future trial and we would investigate how to obtain more timely birth notification.

A challenge relating to the study includes recording the antenatal interaction between the IFH and women. Interestingly, most women asked in Site B were happy for the discussion to be recorded anonymously (i.e. no identifying data was recorded). Concerns were only raised by the IFHs in Site A who did not ask women whether they would be willing for the recording to take place. The recordings provided valuable information about fidelity of delivery. Moving forward to a definitive trial we would recommend that anonymised recording of some interactions take place and that women are specifically asked whether they would agree to this recording on the consent form.

## 5 CONCLUSION

This study has demonstrated that it is feasible to deliver the ABA intervention within an RCT with adequate fidelity. It is feasible to recruit teenagers, women from socioeconomically disadvantaged areas, and women planning to formula feed. Women were willing to be randomised and follow‐up rates were satisfactory. The intervention was acceptable to women, IFHs and maternity services. There is a need for a future definitive trial to test both effectiveness and cost‐effectiveness of the intervention in improving rates of breastfeeding initiation and continuation.

## CONFLICTS OF INTEREST

KJ reports grants from NIHR, local authority funding for the intervention, and part‐funding by NIHR CLAHRC West Midlands during the conduct of the study.

Alongside her Cardiff University role, HT also worked part‐time as a Senior Researcher for NCT charity during the period that the research was conducted. NCT provides breastfeeding peer support services. NCT volunteers were not included in this study.

PH is working on a funding application to take forward the FEST feasibility trial that she led and which is cited in this report. The FEST feasibility trial informed parts of the design of the ABA study.

## CONTRIBUTIONS

JC and JI produced the first draft of this manuscript with input from KJ; all other co‐authors have read and commented on subsequent drafts and have approved the final submitted version. KJ (principal investigator), PH, JI, FD, GT, CM, SD and HT conceived the idea for this research and were involved in the study design and intervention development. Qualitative work and analysis was overseen by GT and conducted by JC, DJ, JI and GT. Statistical analysis was conducted by JC and overseen by AS. SD provided expertise on the use of Behaviour Change Techniques. HT led on the development and analysis of antenatal fidelity checking. MF provided trial expertise. TR provided expertise in health economics.

## Supporting information

Table S1. Characteristics of participants who were followed‐up versus those who did not respond/withdrew (at postnatal text and 8 weeks)
Table S2. Secondary outcomes: maternal wellbeing at 8 weeks and 6 months, satisfaction with home and hospital support for feeding, use of feeding support services at 8‐weeks
