# Supplementary material for: The ABA intervention for improving breastfeeding initiation and continuation: Feasibility study results
Source: Matern Child Nutr. 2019 Dec 2;16(1):e12907. doi: 10.1111/mcn.12907 (PMC7038877; doi:10.1111/mcn.12907)
Supplement: Supplementary file 1 — Table S1. Characteristics of participants who were followed‐up versus those who did not respond/withdrew (at postnatal text and 8 weeks) Table S2. Secondary outcomes: maternal wellbeing at 8 weeks and 6 months, satisfaction with home and hospital support for feeding, use of feeding support services at 8‐weeks [file MCN-16-e12907-s001.docx]

**WEB TABLE 1: Characteristics of participants who were followed-up versus those who did not respond/withdrew (at postnatal text and 8 weeks)**

|  | **Returned postnatal text (baby <14 days)**  **N=70** | **Did not return postnatal text (baby <14 days)^a^**  **N=33** | **Followed up at 8 weeks (returned 8-week questionnaire)**  **N=88** | **Did not return questionnaire at 8 weeks^a^**  **N=14** |
| --- | --- | --- | --- | --- |
| Mean age (years) | 29.8 | 25.7 | 28.7 | 27.1 |
| Ethnicity White British, n/N (%) | 59/70 (84.3) | 29/33 (90.6) | 75/88 (85.2) | 13/14 (92.9) |
| In paid work, n/N (%) | 67/70 (95.7) | 23/33 (71.9) | 79/88 (89.8) | 11/14 (78.6) |
| Educated to degree level or higher, n/N (%) | 39/70 (55.7) | 7/33 (21.9) | 42/88 (47.7) | 4/14 (28.6) |
| Married/civil partnership/living together, n/N (%) | 62/70 (88.6) | 24/33 (77.4) | 75/88 (86.2) | 11/14 (78.6) |
| Feeding intentions (first 6 months), n/N (%) |  |  |  |  |
| Breastmilk only | 28/70 (40.0) | 7/33 (22.6) | 33/88 (37.9) | 2/14 (14.3) |
| Mainly breastmilk | 23/70 (32.9) | 7/33 (22.6) | 25/88 (28.7) | 5/14 (35.7) |
| Half and half | 12/70 (17.1) | 10/33 (32.3) | 19/88 (21.8) | 3/14 (21.4) |
| Mainly formula | 2/70 (2.9) | 3/33 (9.7) | 2/88 (2.3) | 3/14 (21.4) |
| Formula only | 5/70 (7.1) | 4/33 (12.9) | 8/88 (9.2) | 1/14 (7.1) |
| Mean gestational age (weeks) | 39.8 | 39.0^b^ | 39.5 | 40.0^b^ |
| Any breastfeeding at 8 weeks (including health visitor data), n/N (%)^c^ | 41/69 (59.4) | 5/29 (17.2) | 45/88 (51.1) | 1/10 (10.0) |

^a^data not available for one participant who withdrew

^b^no data for stillbirth (n=1)

^c^data not available for 5 participants

**Web-table 2: Secondary outcomes: maternal wellbeing at 8 weeks and 6 months, satisfaction with home and hospital support for feeding, use of feeding support services at 8-weeks**

|  | **Intervention**  **N=50** | **Usual care**  **N=53** | **All**  **N=103** |
| --- | --- | --- | --- |
| Maternal wellbeing WEMWBS  Mean (95% CI), N |  |  |  |
| Change from baseline to 8 weeks | -2.5 (-4.6, -0.5), 40 | -0.1 (-3.1, 3.0), 44 | -1.2 (-3.1, 0.6), 84 |
| Change from baseline to 6 months | -2.9 (-4.8, -1.0), 39 | -0.2 (-2.8, 2.4), 44 | -1.5 (-3.1, 0.1), 83 |
| Maternal satisfaction with hospital support, Mean (95% CI), N | 7.2 (6.3, 8.1), 40 | 7.1 (6.3, 7.9), 42 | 7.2 (6.5, 7.8), 82 |
| Maternal satisfaction with health service support at home Mean (95% CI), N | 7.8 (7.0, 8.6), 40 | 7.7 (7.0, 8.4), 44 | 7.7 (7.2, 8.3), 84 |
| Infant Feeding Counsellor / breastfeeding supporter, n (%) | 20 (51.3) | 7 (16.7) | 27 (33.4) |
| National breastfeeding telephone helpline, n (%) | 2 (5.0) | 2 (4.8) | 4 (4.9) |
| Breastfeeding group, n (%) | 11 (27.5) | 11 (26.2) | 22 (26.8) |
| Internet / web resources, n (%) | 23 (57.5) | 18 (42.9) | 48 (50.0) |
| Social media, n (%) | 8 (20.0) | 6 (14.2) | 14 (17.0) |

^1^WEMWBS = Warwick-Edinburgh Mental Wellbeing Scale^97^ (score ranging from 14-70; 70 indicates highest level of mental wellbeing);

^2^Satisfaction scores – maximum 10.
